# Alpha-Synuclein affects neurite morphology, autophagy, vesicle transport and axonal degeneration in CNS neurons

**DOI:** 10.1038/cddis.2015.169

**Published:** 2015-07-09

**Authors:** J C Koch, F Bitow, J Haack, Z d'Hedouville, J-N Zhang, L Tönges, U Michel, L M A Oliveira, T M Jovin, J Liman, L Tatenhorst, M Bähr, P Lingor

**Affiliations:** 1 Department of Neurology, University Medicine Göttingen, Göttingen, Germany; 2 Lab of Cellular Dynamics, Max Planck Institute for Biophysical Chemistry, Göttingen, Germany; 3 Center for Nanoscale Microscopy and Molecular Physiology of the Brain (CNMPB), Göttingen, Germany

## Abstract

Many neuropathological and experimental studies suggest that the degeneration of dopaminergic terminals and axons precedes the demise of dopaminergic neurons in the substantia nigra, which finally results in the clinical symptoms of Parkinson disease (PD). The mechanisms underlying this early axonal degeneration are, however, still poorly understood. Here, we examined the effects of overexpression of human wildtype alpha-synuclein (*α*Syn-WT), a protein associated with PD, and its mutant variants *α*Syn-A30P and -A53T on neurite morphology and functional parameters in rat primary midbrain neurons (PMN). Moreover, axonal degeneration after overexpression of *α*Syn-WT and -A30P was analyzed by live imaging in the rat optic nerve *in vivo*. We found that overexpression of *α*Syn-WT and of its mutants A30P and A53T impaired neurite outgrowth of PMN and affected neurite branching assessed by Sholl analysis in a variant-dependent manner. Surprisingly, the number of primary neurites per neuron was increased in neurons transfected with *α*Syn. Axonal vesicle transport was examined by live imaging of PMN co-transfected with EGFP-labeled synaptophysin. Overexpression of all *α*Syn variants significantly decreased the number of motile vesicles and decelerated vesicle transport compared with control. Macroautophagic flux in PMN was enhanced by *α*Syn-WT and -A53T but not by *α*Syn-A30P. Correspondingly, colocalization of *α*Syn and the autophagy marker LC3 was reduced for *α*Syn-A30P compared with the other *α*Syn variants. The number of mitochondria colocalizing with LC3 as a marker for mitophagy did not differ among the groups. In the rat optic nerve, both *α*Syn-WT and -A30P accelerated kinetics of acute axonal degeneration following crush lesion as analyzed by *in vivo* live imaging. We conclude that *α*Syn overexpression impairs neurite outgrowth and augments axonal degeneration, whereas axonal vesicle transport and autophagy are severely altered.

Growing evidence suggests that Parkinson's disease (PD) pathology starts at the presynaptic terminals and the distal axons and is then propagated back to the soma in a 'dying back' pattern.^1, 2^ Accordingly, at the time of clinical onset, there is only a 30% loss of total substantia nigra pars compacta neurons but a far more severe loss of striatal dopaminergic markers (70–80%), suggesting that axonal terminals of the nigrostriatal pathway are affected earlier.^1^ It is thus essential to understand the pathomechanisms specifically affecting the axon in PD in order to interfere with early disease progression.

Neurodegeneration in PD is accompanied by the appearance of intraneuronal protein aggregates, denoted Lewy bodies (LBs).^3^ Interestingly, also LB pathology is initially found in the distal axons before becoming evident in the neuronal somata, and dystrophic neurites, so called 'Lewy neurites', outnumber LBs in the early stages of PD.^2, 4, 5^ A main component of LBs is the protein alpha-synuclein (*α*Syn) that is not only widely used as a histopathological marker for PD but is also believed to have a major role in PD pathogenesis.^6, 7^ The importance of *α*Syn is further underlined by the discovery of *α*Syn point mutations (e.g. Ala53Thr (A53T), Ala30Pro (A30P)) and multiplications of the *α*Syn gene, all of which cause autosomal dominant forms of PD.^8, 9, 10^ However, neither the physiological functions nor the pathogenetic mechanisms of *α*Syn are well understood.^7^

The biological effects of *α*Syn expression strongly depend on the model system. Wild-type (WT) human *α*Syn does not lead to major clinical or histological abnormalities when expressed in transgenic mice,^11, 12^ but its overexpression mediated by adeno-associated viral vectors (AAV) results in severe neurodegeneration, suggesting a dose-dependent toxic effect.^13, 14^ Different human *α*Syn-A30P and -A53T transgenic mouse lines develop severe motor impairments, partly resembling symptoms of human PD, accompanied by a degeneration of the nigrostriatal neuronal system and LB-like pathology.^11, 12, 15^ In line with the pathological findings in human PD, the axonal compartment is affected early and most prominently in these animal models.

Different putative pathomechanisms of *α*Syn toxicity have been explored. For example, the cytoskeleton is an important molecular target of *α*Syn. Multimeric forms of *α*Syn were shown to impair the polymerization of tubulin and microtubule formation.^16, 17^ Overexpression of *α*Syn increased actin instability and induced actin bundling in cultured hippocampal neurons.^18^ There are, however, divergent data on the resulting effects of *α*Syn overexpression on neurite outgrowth and integrity in different model systems.^19, 20, 21, 22^

Moreover, a dysregulation of autophagy has been implicated in PD pathology. Aberrant *α*Syn is normally degraded by autophagy and only to a negligible degree by the proteasome.^23^ Several studies have shown that the inhibition of autophagy results in an accumulation and increased toxicity of *α*Syn, whereas the activation of autophagy has therapeutic effects in PD models.^23, 24, 25, 26^ However, the direct effects of *α*Syn and its mutants on autophagy seem to rely strongly on the model system and the published data are highly controversial.^24, 26, 27, 28, 29, 30, 31, 32^

Given the central role of axonal degeneration in PD, it is likely that disturbances of axonal transport are involved.^33^ In support of this proposition, the motor protein kinesin was shown to be decreased early and stage-dependently in PD patients, preceding the loss of substantia nigra neurons.^34^ *α*Syn itself is actively transported along the axons, mainly by the slow component of axonal transport, but the role of *α*Syn in axonal vesicle transport is unclear.^35^

Here, we present a comprehensive analysis of the effects of *α*Syn on neurite morphology and examine important pathomechanisms.

## Results

### Effects of *α*-synuclein overexpression on neurite morphology in PMN

**Figure 1:**
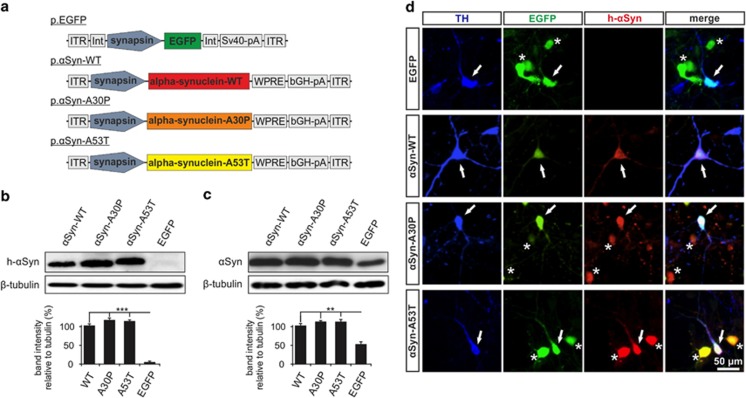
Overexpression of *α*Syn variants in PMN. (**a**) Vector maps of the plasmids used to overexpress EGFP, *α*Syn-WT, -A30P and -A53T. The respective transcripts are expressed under the control of a human synapsin-1 promoter. ITR: AAV-2 inverted terminal repeat. Int: intron. SV40-pA: SV40 polyadenylation site. WPRE: Woodchuck hepatitis virus posttranscriptional regulatory element. bGH-pA: bovine growth hormone polyadenylation site. (**b** and **c**) Immunoblots of whole cell protein lysates from PMN transfected with the given plasmids (cells were lysed on DIV 5). In **b**, an antibody specific for human *α*Syn (LB509, Invitrogen) was used to detect only the *α*Syn expressed by the plasmids. In **c**, total cellular *α*Syn levels were assessed using an antibody recognizing both human and rat *α*Syn (BD). At the bottom, quantifications of the band intensities normalized to *β*-tubulin are shown (*n*=3; error bars represent means±S.E.M.; **P<0.005, ***P<0.0005 according to one-way ANOVA and Dunnett's posthoc test). (**d**) Immunocytochemistry of PMN transfected with the plasmids given on the left side and stained against tyrosine hydroxylase (TH) to identify dopaminergic neurons and human *α*Syn (LB509, Invitrogen) to check for successful transfection with the respective plasmids (micrographs taken on DIV 5). EGFP is expressed by the plasmid p.EGFP that is either transfected alone or co-transfected with the *α*Syn-plasmids. Arrows highlight transfected dopaminergic neurons, asterisks mark transfected non-dopaminergic neurons

To analyze the effects of increased intraneuronal *α*Syn levels on neurite morphology, we transfected PMN with plasmids expressing human *α*Syn-wild-type (p.*α*Syn-WT) or one of the two human *α*Syn-mutants A30P (p.*α*Syn-A30P) and A53T (p.*α*Syn-A53T) (Figure 1). As control, PMN were transfected with a plasmid expressing EGFP (p.EGFP) only. Measurements were performed separately in dopaminergic, that is, tyrosine-hydroxylase (TH)-positive, and non-dopaminergic, that is, TH-negative neurons, to assess differential vulnerability of these neuronal cell types.

**Figure 2:**
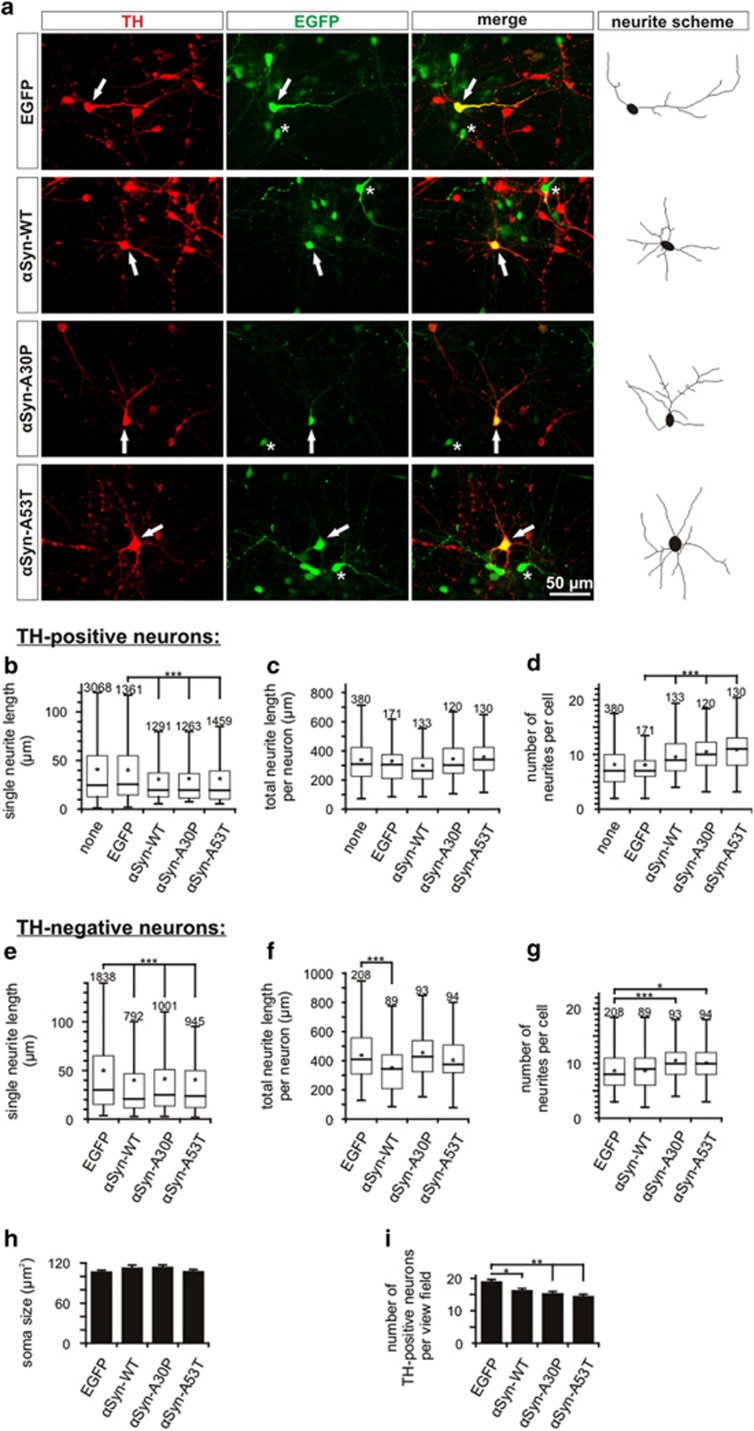
Neurite morphology of PMN transfected with different *α*Syn variants. (**a**) Representative micrographs from an immunocytochemistry of PMN transfected with the plasmids given on the left side and stained against tyrosine hydroxylase (TH); photos were taken on DIV 5. Arrows point at transfected (EGFP positive) dopaminergic (TH-positive) neurons that are also drawn in the neurite scheme on the right side. Asterisks mark transfected non-dopaminergic neurons. (**b–g**) Quantification of single neurite lengths (**b** and **e**), total neurite length per neuron (**c** and **f**) and number of primary neurites per neuron (**d** and **g**) of TH-positive neurons (**b**–**d**) and TH-negative neurons (**e**–**g**) transfected with the given plasmids (DIV 5). The data are shown in box plots (box: range from first to third quartile; band inside box: median (=second quartile); star: arithmetic mean value; bottom end of whiskers: minimum of all data; top end of whiskers: 1.5 interquartile range (IQR) of the upper quartile; number given above the upper whisker: number of single values (*n*) included in the respective quantification). (**h**) Quantification of the soma size of PMN transfected with the given plasmids. (**i**) Quantification of the number of TH-positive neurons per view field ( × 10) on DIV 5 after transfection with the given plasmids. Total neuron numbers did not differ significantly among the groups. Statistics: one-way ANOVA followed by Dunnett's *post hoc* test, *P<0.05, **P<0.005, ***P<0.0005. Error bars represent means±S.E.M

The mean single neurite length was significantly reduced in *TH-positive neurons* overexpressing *α*Syn-WT (34±0.7 *μ*m), *α*Syn-A30P (33±1.0 *μ*m) and *α*Syn-A53T (33±0.8 *μ*m) as compared with EGFP transfected (45±1.4 *μ*m) and untransfected neurons (45±0.9 *μ*m) (Figure 2b). There was no significant difference between the different *α*Syn variants. The same effect was seen in *TH*-*negative neurons* where overexpression of all *α*Syn variants resulted in a significant reduction of the mean single neurite length compared with controls (EGFP: 53±1.2 *μ*m; *α*Syn-WT: 42±1.8 *μ*m; *α*Syn-A30P: 45±1.4 *μ*m; *α*Syn-A53T: 47±1.8 *μ*m) (Figure 2e). Again, there was no significant difference between the different *α*Syn variants.

The mean total length of the complete neurite tree per *TH-positive neuron* did not differ significantly among the groups, but there was a reduced (by ~10%) mean total neurite length by trend in the *α*Syn-WT group (untransfected: 336±7 *μ*m; EGFP: 329±10 *μ*m; *α*Syn-WT: 298±11 *μ*m; *α*Syn-A30P: 337±13 *μ*m; *α*Syn-A53T: 357±11.69 *μ*m) (Figure 2c). A similar result was observed for *TH-negative neurons* where the *α*Syn-WT showed a significantly reduced mean total length of all neuritic processes compared to the other groups (EGFP: 440±12 *μ*m; *α*Syn-WT: 358±18 *μ*m; *α*Syn-A30P: 456±20 *μ*m; *α*Syn-A53T: 414±17 *μ*m) (Figure 2f).

Unexpectedly, the number of neurites originating from the soma was significantly increased in *TH-positive neurons* overexpressing *α*Syn variants (*α*Syn-WT: 9.6±0.3; *α*Syn-A30P: 10.5±0.4; A53T: 11.0±0.31 neurites per neuron) as compared with TH-positive neurons transfected with EGFP only (8.0±0.3 neurites per neuron) and untransfected TH-positive neurons (8.1±0.2 neurites per neuron) (Figure 2d). In *TH-negative neurons*, only the two mutants *α*Syn-A30P and A53T showed a significantly increased neurite number per neuron compared with EGFP control, while there was no difference between *α*Syn-WT and EGFP transfected neurons (EGFP: 8.8±0,3; *α*Syn-WT: 8.9±0.4; *α*Syn-A30P: 10.8±0.5; *α*Syn-A53T: 10.1±0.4 neurites per neuron) (Figure 2g).

There were no differences in the mean soma size of *TH-positive neurons* among the experimental groups (EGFP: 107±2 *μ*m^2^; *α*Syn-WT: 112±2 *μ*m^2^; *α*Syn-A30P: 114±2 *μ*m^2^; *α*Syn-A53T: 107±2 *μ*m^2^) (Figure 2h).

To assess putative effects of *α*Syn-overexpression on cell viability, the cell number of *TH-positive neurons* was compared on day *in vitro* (DIV) 5 (Figure 2i). Compared with the EGFP control, the number of TH-positive neurons was significantly reduced in the groups overexpressing *α*Syn-WT (85±3%) and even further reduced for A30P (81±3%) or A53T (76±3%). Total neuron numbers did not differ significantly among the groups, suggesting a special vulnerability of the TH-positive neurons to *α*Syn toxicity.

**Figure 3:**
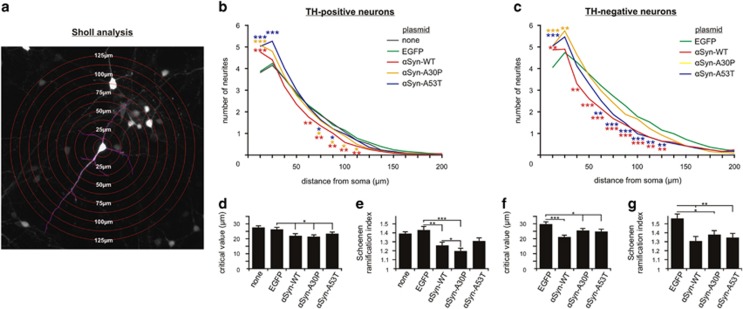
Sholl analysis of PMN transfected with different *α*Syn variants. (**a**) Representative micrograph of an EGFP-transfected TH-positive neuron with highlighted neurites (purple) and superimposed circles at given distances from the center of the soma. For Sholl analysis, the total number of neurite crossings was counted at each circle with the radius increasing in steps of 12.5 *μ*m. (**b** and **c**) Results of the Sholl analysis of TH-positive-neurons (**b**) and TH-negative neurons (**c**) transfected with the given plasmids (DIV 5). The mean number of neurite crossings at a given distance from the center of the soma is plotted. (**d–g**) Additional quantifications of the Sholl analysis of TH-positive neurons (**d** and **e**) and TH-negative neurons (**f** and **g**) transfected with the given plasmids (DIV 5). The critical value (**d** and **f**) is the radius *r* at which there is a maximum number of neurite crossings ('neurite maximum'). The Schoenen ramification index (**e** and **g**) is calculated by dividing the neurite maximum (i.e. the maximum number of branches as specified by the critical value) by the number of primary neurites originating from the soma. It represents the degree of ramification of a neurite tree. Statistics: one-way ANOVA followed by Dunnett's *post hoc* test, *P<0.05, **P<0.005, ***P<0.0005. Error bars represent means±S.E.M. *n*=135 randomly chosen neurons per condition from three independent experiments

Next, a Sholl analysis was performed of the transfected PMN to assess the effects of *α*Syn overexpression on neuritic branching behavior.^36^ As shown in Figure 3, the branching behavior of the neurons transfected with *α*Syn variants clearly differed from the EGFP-transfected and the untransfected neurons. The critical value, the radius *r* at which there is a maximum number of neurite crossings ('neurite maximum'), was significantly lower in the *α*Syn-transfected groups as compared with controls (*TH-positive neurons:* untransfected: 28±1 *μ*m; EGFP: 26±1 *μ*m; *α*Syn-WT: 22±1 *μ*m; *α*Syn-A30P: 22±1 *μ*m; *α*Syn-A53T: 23±1 *μ*m; *TH-negative* *neurons:* EGFP: 30±2 *μ*m; *α*Syn-WT: 21±2 *μ*m; *α*Syn-A30P: 25±2 *μ*m; *α*Syn-A53T: 25±2 *μ*m) (Figures 3d and f). That is, the point of maximum neurite branching was shifted closer to the soma in the *α*Syn-overexpressing neurons. The Schoenen ramification index represents the degree of ramification of a neurite and is calculated by dividing the neurite maximum (i.e. the maximum number of branches as specified by the critical value) by the number of primary neurites originating from the soma.^37^ It was significantly lower compared with EGFP control for *α*Syn-WT and -A30P in TH-positive neurons and for *α*Syn-A30P and A53T in TH-negative neurons (*TH-positive neurons:* untransfected: 1.39±0.02; EGFP: 1.43±0.05; *α*Syn-WT: 1.26±0.04; *α*Syn-A30P: 1.20±0.03; *α*Syn-A53T: 1.28±0.04. *TH-negative neurons:* EGFP: 1.56±0.05; *α*Syn-WT: 1.31±0.05; *α*Syn-A30P: 1.38±0.05; *α*Syn-A53T: 1.35±0.05) (Figures 3e and g). This result implies that *α*Syn overexpression impairs neurite branching in both dopaminergic and non-dopaminergic neurons.

### *α*-Synuclein variants differently affect macroautophagy in PMN

**Figure 4:**
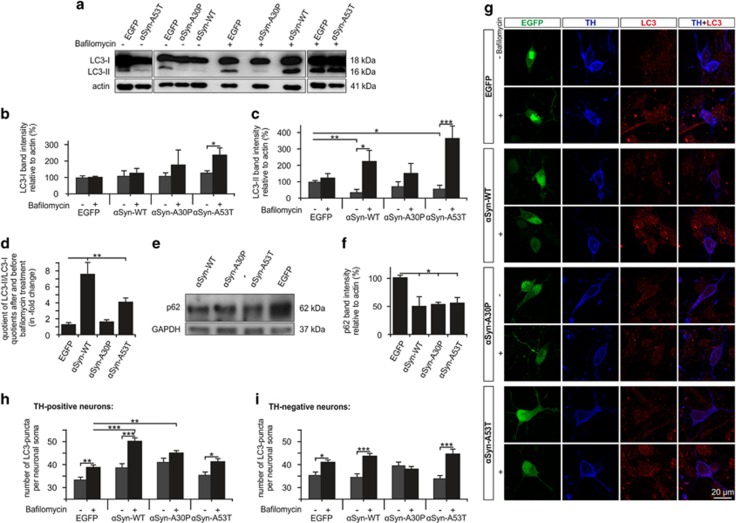
Macroautophagy in PMN transfected with different *α*Syn variants. (**a**) Representative LC3 immunoblots of whole cell protein lysates from PMN transfected with the plasmids given above. As indicated above the blot, the groups on the right side were treated with bafilomycin (1 nM for 6 h) for evaluation of autophagic flux. The photo represents optimal exposition times for detection of the LC3-II band; for quantification of the higher intensity LC3-I band, photographs with lower exposition times were chosen. (**b–d**) Quantifications of the band intensities normalized to actin as loading control are shown for LC3-I (**b**) and LC3-II (**c**). Changes in autophagic flux are displayed in **d**, where the quotients of the LC3-II/LC3-I quotients after and before bafilomycin treatment are shown for each given plasmid. (**e** and **f**) Representative p62-immunoblot (**e**) of whole cell protein lysates from PMN transfected with the plasmids given above. Quantifications of the band intensities normalized to actin as loading control are shown in **f**. (**g**) Representative pseudo-confocal micrographs from an immunocytochemistry of PMN transfected with the plasmids given on the left side, with (+) or without (−) bafilomycin treatment as indicated on the left side and stained against tyrosine hydroxylase (TH) and LC3; photos were taken on DIV 5. (**h** and **i**) Quantification of the LC3 immunocytochemistry (representative micrographs: see **g**). Intraneuronal LC3 puncta were counted in TH-positive neurons (**h**) and TH-negative neurons (**i**) transfected with the given plasmids with (+) or without (−) bafilomycin treatment on pseudo-confocal micrographs (DIV 5). Statistics: one-way ANOVA followed by Dunnett's *post hoc* test, *P<0.05, **P<0.005, ***P<0.0005. Error bars represent means±S.E.M. *n*=3 independent experiments

Immunoblot analysis of PMN protein lysates was performed to assess the expression levels of the two isoforms of the microtubule-associated protein 1 light chain 3 (LC3): LC3-I (18 kDa) and its PE-conjugated form LC3-II (16 kDa) (Figure 4a). For evaluation of autophagic flux, cells were treated with the vacuolar-type H^+^-ATPase inhibitor bafilomycin A1, which arrests autophagy at the lysosomal level and thereby unmasks the transit of LC3-II through the autophagic pathway.^38^ All conditions showed similar expression levels of LC3-I except for the bafilomycin-treated *α*Syn-A53T group that displayed a mild increase of LC3-I (Figure 4b). The basal levels of LC3-II (without bafilomycin) were significantly reduced in PMN overexpressing *α*Syn-WT and -A53T as compared with EGFP control (Figure 4c). After bafilomycin treatment, however, there was a strong increase in LC3-II levels in the neurons transfected with *α*Syn-WT and -A53T that was not as pronounced in the EGFP control and *α*Syn-A30P groups (Figure 4c). To visualize the changes in autophagic flux, the LC3-II/LC3-I quotient with bafilomycin treatment was divided by the respective quotient without bafilomycin for each transfection group. The resulting quotient was significantly increased compared with EGFP control for both *α*Syn-WT and -A53T but not for *α*Syn-A30P (Figure 4d).

SQSTM1/p62 (p62) serves as a link between LC3 and ubiquitinated substrates and is an indirect marker for autophagic activity.^38^ We found significantly decreased expression levels for all *α*Syn variants compared with control as assessed by immunoblotting of PMN protein lysates (Figures 4e and f).

To confirm and further specify the immunoblot results, we performed an LC3 immunocytochemistry (ICC) of transfected PMN (Figure 4g) and quantified the number of LC3 puncta per neuronal soma, which correlates to the amount of intracellular autophagosomes.^38^ In TH-positive neurons (Figure 4h), total numbers of LC3 puncta were higher for all *α*Syn variants compared with EGFP. After bafilomycin treatment, the number of LC3 puncta was significantly increased for *α*Syn-WT, -A53T and EGFP while there was no increase for *α*Syn-A30P. The increase of LC3 puncta was significantly more pronounced in the *α*Syn-WT transfected neurons. In TH-negative neurons (Figure 4i), the increase of LC3 puncta after treatment with bafilomycin was significantly more pronounced in both *α*Syn-WT and -A53T transfected neurons as compared with EGFP control. For *α*Syn-A30P, the basal number of LC3 puncta was significantly higher compared with the other groups but there was no significant change in the numbers of LC3 puncta after bafilomycin treatment.

**Figure 5:**
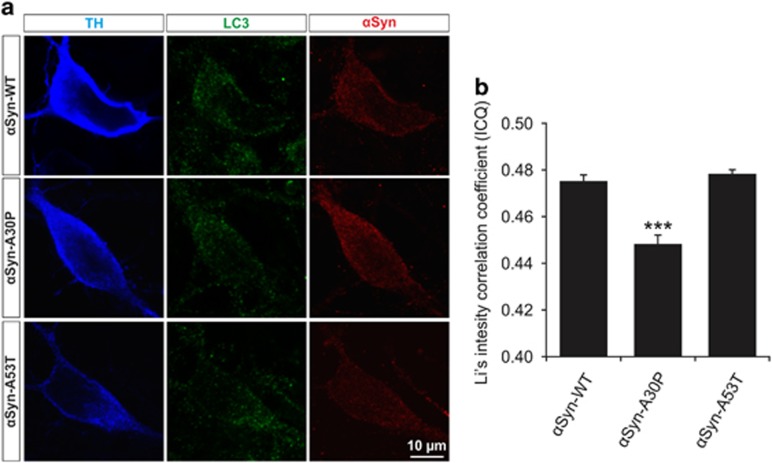
Colocalization of LC3 and *α*Syn in PMN transfected with different *α*Syn variants. (**a**) Representative confocal micrographs of PMN transfected with the respective *α*Syn variant given on the left side and immunostained against TH, LC3 and *α*Syn as specified on top. (**b**) Quantification of colocalization between *α*Syn and LC3 in the soma of TH-positive neurons after transfection with different *α*Syn variants represented by means of Li's intensity correlation coefficient. Cells transfected with *α*Syn-A30P show significantly less colocalization than those transfected with *α*Syn-WT or -A53T. Statistics: one-way ANOVA followed by Tukey-Kramer *post hoc* test, ***P<0.0005 as compared with both other groups. Error bars represent means±S.E.M. *n*=2 independent experiments

In order to establish whether differences in autophagy are due to altered binding of *α*Syn to autophagosomes, we evaluated the colocalization of *α*Syn and LC3 in transfected PMN by confocal microscopy (Figure 5). Similar to the LC3 staining, the *α*Syn staining showed a punctuate pattern in soma and neurites. Calculation of Li's intensity correlation coefficient^39, 40^ confirmed a high level of colocalization between *α*Syn and LC3, which, however, was significantly reduced for *α*Syn-A30P (*α*Syn-WT: 0.475±0.003; *α*Syn-A30P: 0.448±0.002; *α*Syn-A53T: 0.478±0.002) (Figure 5b).

To assess the putative effects of *α*Syn variants on the autophagic degradation of mitochondria (mitophagy), PMN were transduced with AAV expressing different *α*Syn variants. After ICC for LC3 and the mitochondrial marker TOM20,^41^ the number of mitochondria colocalizing with LC3 was quantified in TH-positive neurons using confocal microscopy. The ratio of LC3 puncta colocalizing with TOM20 divided by the total number of LC3 puncta did not differ significantly between the groups (EGFP: 0.102±0.005; *α*Syn-WT: 0.101±0.005; *α*Syn-A30P: 0.102±0.005; *α*Syn-A53T: 0.093±0.005) (Supplementary Figure 1).

### Transport of synaptophysin vesicles is impaired by overexpression of *α*-synuclein variants

**Figure 6:**
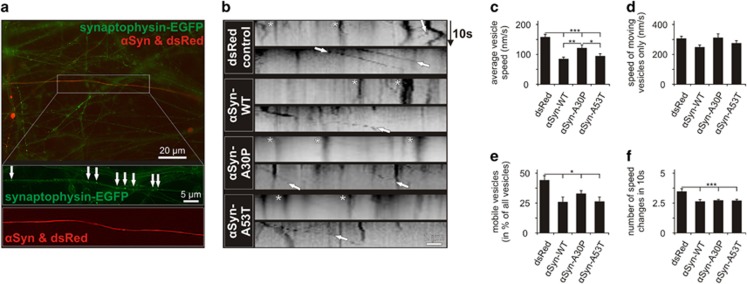
Axonal transport of synaptophysin-positive vesicles in PMN transfected with different *α*Syn variants. (**a**) Representative micrograph showing PMN transduced with AAV.synaptophysin-EGFP (expressing EGFP-tagged synaptophysin) and co-transfected with p.*α*Syn-WT and p.dsRed (to identify transfected neurons) (DIV 5). In the higher magnification pictures, the EGFP-positive vesicles (arrows) can be seen along the transfected axon. (**b**) Representative kymographs of the movements of EGFP-tagged synaptophysin along neurites transduced with the plasmids given on the left side within 10 s (*y* axis). Arrows point at representative moving vesicles, asterisks mark stationary vesicles. (**c–f**) Quantifications of synaptophysin-EGFP transport in neurites transfected with the given plasmids. Statistics: one-way ANOVA followed by Dunnett's *post hoc* test, *P<0.05, **P<0.005, ***P<0.0005. Error bars represent means±S.E.M. *n*=3 independent experiments

To analyze the influence of *α*Syn overexpression on the transport of synaptic vesicles, we produced AAV expressing synaptophysin tagged with EGFP. Synaptophysin is actively transported in vesicles along the axon by fast axonal transport.^42^ PMN were co-transfected with plasmids overexpressing *α*Syn variants, a plasmid expressing dsRed (to allow identification of transfected neurons) and AAV.synaptophysin-EGFP. On DIV 5, live imaging of the movements of EGFP-labeled synaptophysin vesicles in transfected neurons was performed and kymographs of single neurites were reconstructed (Figure 6). The mean velocity of all vesicles was significantly reduced in the *α*Syn variants groups compared with EGFP, although to a lower degree by *α*Syn-A30P (Figure 6c). This effect was mainly caused by a significantly lower percentage of moving vesicles in all *α*Syn variants groups (Figure 6e). The velocity of the moving vesicles was only mildly reduced for *α*Syn-WT compared with control but not affected by the other *α*Syn variants (Figure 6d). Moreover, the number of speed changes was significantly reduced in all *α*Syn variants groups compared with control (Figure 6f).

### *α*-Synuclein enhances axonal degeneration after optic nerve crush lesion *in vivo*

**Figure 7:**
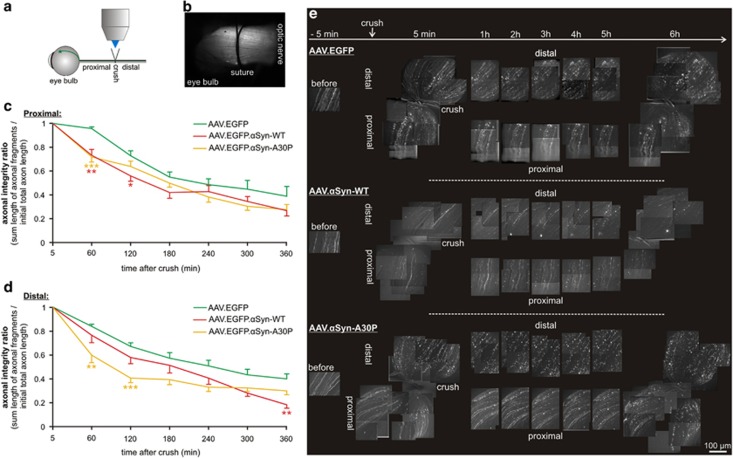
*In vivo* live imaging of acute axonal degeneration in the rat optic nerve. (**a**) Schematic drawing of the experimental setup. The optic nerve was crushed with a suture and then imaged *in vivo* on the proximal and distal side of the crush for 6 h. Single axons from retinal ganglion cells were labeled with EGFP expressed by AAV that were injected intravitreally 4 weeks before the imaging. (**b**) Low magnification micrograph of an exposed optic nerve before crush lesion. The suture is loosely tied around the nerve. Single EGFP-positive axons can be identified. (**c** and **d**) Quantification of the AIR at the given time-points after crush lesion on the proximal (**c**) and distal side (**d**) of the crush (*n*=15 axons from three independent experiments per group). Statistics: one-way ANOVA followed by Dunnett's *post hoc* test, *P<0.05, **P<0.005, ***P<0.0005. Error bars represent means±S.E.M. (**e**) Representative composite images of projected z-stack micrographs of optic nerves transduced with AAV.EGFP, AAV.*α*Syn-WT or AAV.*α*Syn-A30P at the time points after crush lesion given on top

Next, we analyzed the effects of *α*Syn overexpression on axonal degeneration *in vivo*. As a model system, we chose the well-established rat optic nerve crush that leads to a reproducible fragmentation of the axon adjacent to the crush site that can be monitored by *in vivo* live imaging.^43, 44, 45^ AAV conferring overexpression of *α*Syn-WT or -A30P and EGFP were injected intravitreally 4 weeks before the imaging to allow for sufficient expression of the transcript in retinal ganglion cell axons (Supplementary Figure 2).^46^ An optic nerve crush was performed and the area 1 mm around the crush site was imaged over 6 h. For each time point, we determined the mean axonal integrity ratio (AIR), defined as the sum length of axonal fragments at a given time point divided by the initial total axon length before fragmentation.^44^ On the proximal side of the crush, both AAV.*α*Syn-WT and AAV.*α*Syn-A30P significantly enhanced axonal degeneration as reflected by a faster decrease of the AIR at 60 min and 120 min after crush compared with control animals that had been injected with AAV.EGFP (Figure 7c) (AIR at 60 min: EGFP control: 0.96±0.01, *α*Syn-WT: 0.73±0.05, *α*Syn-A30P: 0.71±0.04; at 120 min: EGFP: 0.73±0.04, *α*Syn-WT: 0.56±0.04, *α*Syn-A30P: 0.64±0.05). At later time points after crush, the axonal integrity remained at lower levels in the optic nerves transduced with *α*Syn-WT and *α*Syn-A30P although the difference was not significant. On the distal side, *α*Syn-A30P strongly enhanced axonal degeneration at the early time points, whereas *α*Syn-WT significantly decreased axonal integrity at 6 h after crush (AIR at 60 min: EGFP: 0.84±0.02, *α*Syn-WT: 0.77±0.07, *α*Syn-A30P: 0.60±0.07; at 120 min: EGFP: 0.67±0.03, *α*Syn-WT: 0.58±0.06, *α*Syn-A30P: 0.41±0.04; at 360 min: EGFP: 0.40±0.04, *α*Syn-WT: 0.19±0.03, *α*Syn-A30P: 0.30±0.03).

## Discussion

The protein *α*Syn is believed to have a central pathogenic role in PD while degeneration of dopaminergic axons is one of the initial pathological events observed. We therefore studied the role of *α*Syn in several cellular processes that are essential for maintenance of neurite integrity.

Overexpression of *α*Syn in PMN results in decreased neurite length. These data support and expand previous studies that reported a reduced neurite outgrowth after *α*Syn-WT overexpression in B103 cells,^19^ transfection of primary hippocampal neurons with *α*Syn-WT and -A30P,^18^ and treatment of rat cortical neurons with *α*Syn oligomers.^20^ Lui *et al.*^22^ however, reported an increased neurite outgrowth in rat cortical neurons after treatment with *α*Syn-WT and -mutant oligomers and in MES23.5 dopaminergic neurons expressing *α*Syn-WT. This may represent an acute reaction of the neurons, which was observed only 4–24 h after seeding, in contrast to the more chronic *α*Syn overexpression paradigm used in our study. Interestingly, *α*Syn appears to impair neurite elongation but not initiation of neurite growth because the number of primary neurites was increased in PMN. Whereas neurite initiation is highly actin-dependent, neurite elongation requires tubulin polymerization. Several publications have reported that *α*Syn inhibits tubulin polymerization^17^ and also associates with the actin cytoskeleton, exerting an actin-bundling activity.^18^ Our data thus yield a morphological correlate to these interactions of *α*Syn with tubulin and actin.

The neurite elongation deficiency is further reflected in the reduced ramification of the neurite tree as assessed by Sholl analysis. Interestingly, LB pathology preferentially affects hyperbranched neurites in human brain tissue.^47^ The PMN in our study do not develop LBs, but elevated *α*Syn levels are deleterious to higher degree neurite branching also in our model.

In our paradigm, the effects on neurite morphology were less dependent on the transfected *α*Syn variant, but rather on *α*Syn overexpression in general, corresponding well to the dose-dependent toxicity of *α*Syn in human *α*Syn gene multiplications.^7, 10^ It is noteworthy that the effects of *α*Syn on neurite integrity became obvious with only a mild degree of overexpression and a relatively short observation period of 5 days, emphasizing the relevance of this pathomechanism.

In contrast, we observed markedly differential effects of the *α*Syn variants on autophagy. Although a number of studies report the effects of *α*Syn on autophagy in different cell models with largely contradictory results, this is, to our knowledge, the first comprehensive study comparing all relevant parameters of macroautophagy for *α*Syn-WT, -A53T and -A30P in PMN. In accordance with previous reports from rat cortical neurons^30^ and SHSY5Y cells,^31^ we found an increased autophagic flux in the *α*Syn-WT and -A53T transfected PMN. Increased markers of macroautophagy have been reported in human sporadic PD and A53T-mutant brains.^48^ However, large evidence suggests that autophagic flux is decreased in PD and that induction of autophagy might have therapeutic effects.^25^ In *α*Syn-WT transgenic mice, it was demonstrated that macroautophagy is activated depending on the *α*Syn burden.^32^ Therefore, the increased autophagic flux in the *α*Syn-WT- and -A53T-transfected PMN in our study likely represents a physiological response to increased *α*Syn levels. However, increased autophagy might also foster the unspecific degradation of essential proteins or organelles and thereby contribute, at least partly, to pathology.

Effects of *α*Syn-A30P on macroautophagy in PMN have not been described before. We show here, that PMN expressing *α*Syn-A30P were not able to promote autophagic flux in response to the increased *α*Syn burden. This inhibition of autophagic flux could be a central pathomechanism of A30P toxicity. A possible explanation is the disrupted binding of *α*Syn-A30P to membranes that could impair interactions of *α*Syn with autophagosomes.^49^ In favor of this explanation, we found a decreased colocalization of *α*Syn and LC3 in dopaminergic neurons overexpressing *α*Syn-A30P.

Specific effects of *α*Syn on the autophagic degradation of mitochondria (mitophagy) have not been studied sufficiently, although disturbed mitochondrial function by *α*Syn overexpression was demonstrated^50^ and other PD-causing mutations result in impaired mitophagy.^41^ Increased numbers of mitochondria colocalizing with macroautophagic markers were reported in transgenic mice overexpressing *α*Syn-A53T, yet the conclusions on the state of mitophagy were contradictory.^30, 51, 52^ Here, we did not detect any significant effects of *α*Syn overexpression on the number of mitochondria colocalizing with LC3. This suggests that mitophagy is regulated independently of general macroautophagy under our experimental settings.

Axonal transport is pivotal for the maintenance of neurite integrity. We demonstrate here for the first time, that *α*Syn overexpression impairs fast axonal transport of synaptic vesicles. PMN-overexpressing *α*Syn variants had a reduced number of moving vesicles and these showed less speed changes. It was reported before that *α*Syn itself is actively transported along the axon.^35^ Inconsistent data have been published with regard to the effects of the mutant forms A30P and A53T on *α*Syn transport velocity.^53, 54, 55^ However, these studies neither examined transport of other cargoes nor did they include controls different from *α*Syn-WT, so possible effects of *α*Syn overexpression were not assessed. Histological analysis of human brain tissue showed that the levels of the motor protein kinesin are reduced early in PD^34^ and that LBs contain axonally transported proteins like synphilin and synaptophysin.^56^ In rat cortical neurons, a co-immunoprecipitation of *α*Syn with the motor proteins kinesin and dynein was demonstrated.^57^ A possible explanation for the impairment of axonal transport is thus an inhibition or sequestration of motor proteins by *α*Syn. As axonal transport depends on intact microtubules,^33^ defects in tubulin polymerization induced by *α*Syn could also contribute to impaired axonal transport.^17^

In the rat optic nerve crush model, we found an accelerated axonal degeneration in axons overexpressing *α*Syn, confirming the specific detrimental effects of *α*Syn overexpression on axonal integrity *in vivo*. Supporting our results, mice overexpressing human *α*Syn-WT have increased signs of axonal degeneration in the peripheral nervous system.^58^ The enhanced axonal degeneration is likely to be linked to pathomechanisms that we have explored *in vitro*: increased autophagy and disturbed axonal transport both enhance axonal degeneration.^33, 43^

Interestingly, differential effects of the *α*Syn mutants A30P or A53T were only found on macroautophagy, whereas neurite outgrowth and axonal transport were equally impaired by increased intraneuronal *α*Syn levels independent of the specific *α*Syn variant. On the basis of our findings, the A30P mutation might exert its specific toxicity by impairing autophagy and thereby indirectly increasing intraneuronal *α*Syn levels among other detrimental consequences of decreased autophagy. The specific differential pathomechanism of the A53T mutant could not be further discriminated in our study. It has been shown before that *α*Syn-A53T impairs mitochondrial function and increases ROS production, which could affect intraneuronal degradation pathways on a longer time scale.^59^ In a parallel study, we found that both *α*Syn-A30P and -A53T but not -WT impaired regeneration of lesioned dopaminergic axons *in vitro* and *in vivo*.^60^ Again, this shows that the mutations become relevant under stress conditions over time.

In summary, we have demonstrated that mild overexpression of *α*Syn in PMN impairs neurite outgrowth, neurite ramification and axonal vesicle transport equally for the wild-type protein and the familial A30P and A53T mutant forms. In contrast, the mutant *α*Syn-A30P specifically inhibits autophagic flux, whereas *α*Syn-WT and -A53T both increase macroautophagy. *In vivo*, axonal degeneration is enhanced after *α*Syn overexpression. These data characterize elevated intraneuronal levels of *α*Syn as a detrimental factor for neurite integrity and present several *α*Syn-controlled intracellular processes that contribute to *α*Syn-mediated pathophysiology and may represent promising therapeutic targets.

## Materials and Methods

### Plasmids and AAV

For *in vitro* experiments in PMN, the following plasmids were used: Control Plasmids expressing EGFP (p.EGFP) or dsRed (p.dsRed) under control of a human synapsin-1 promoter and containing a simian vacuolating virus 40 polyadenylation (Sv40-pA) sequence to enhance transcription as described previously (Gen-Bank ID: HQ416702 & AY640633).^61^ Plasmids expressing *α*Syn variants (p.*α*Syn-WT, p.*α*Syn-A30P, p.*α*Syn-A53T) were a gift from Grit Taschenberger, Manuel Garrido and Sebastian Kügler (Göttingen, Germany) and have been described elsewhere,^62^ expression of *α*Syn variants was driven by a human synapsin-1 promoter and enhanced by a Woodchuck hepatitis virus posttranscriptional regulatory element (WPRE) and a bovine growth hormone polyadenylation site (bGH-pA) (Figure 1a). The *α*Syn-A53T was tagged with a UA-tag.

Axonal transport in PMN was visualized by transduction with an AAV expressing synaptophysin-EGFP. For cloning of pAAV.synaptophysin-EGFP, the previously described pMH4-I-Syn-p38(=rat synaptophysin)-EGFP^42^ (generous gift from Asparouh I. Iliev, now at University of Bern) was cut with *Sma*I and *Xho*I. The resulting synaptophysin-EGFP fragment was cloned into an *Eco*RV- and *Xho*I-cut pAAV-chicken-*β*-actin-CMV-enhancer-MCS-WPRE-bGH (from Uwe Michel, Göttingen, Germany). The resulting pAAV-chicken-*β*-actin-CMV-enhancer-synaptophysin-EGFP-WPRE-bGH was used for AAV production as described below.

For *in vivo* experiments, AAV overexpressing *α*Syn-WT and -A30P were used. They were produced on the basis of the plasmids pAAV-noTB-SEIS+aSyn-WT-SWBnew (for *α*Syn-WT) and pAAV-noTB-aSyn-A30P-with-SmaI+SEIS (for *α*Syn-A30P), both generous gifts from Manuel Garrido. Both plasmids express either *α*Syn-WT or -A30P under control of a human synapsin-1 promoter enhanced by a WPRE and a bGH-pA site and independently co-express EGFP driven by a second human synapsin-1 promoter enhanced by a Sv40-pA site. As control for the *in vivo* experiments, the previously described AAV-9(5)hSyn-EGFP-CytbAS-ohneNot was used.^61^ All plasmids were sequenced to confirm their correct sequence.

Production of AAV (hybrid serotype 2/1) was performed as described previously.^63^ Briefly, 293-HEK cells were transfected with calcium phosphate, HEPES-buffered saline and a serotype specific plasmid mix (pAAV-RC, pH21 (gift from Helen Fitzsimons (Neurologix, Inc. OSU Comprehensive Cancer Center, Columbus, OH, USA) and Matthew During (Molecular Virology, Immunology, and Medical Genetics, Columbus, OH, USA), pHELPER and the respective pAAV expression vector in a 0.5:0.5:1:1 molar ratio)). Cells were harvested 48 h after transfection and AAV were purified by dialysis and virus gradient centrifugation in iodixanol. Fast protein liquid chromatography was performed to obtain high titer viral stocks (titers: 2–4 × 10^8^ TU/*μ*l). The virus stocks were then tested on primary cortical neurons for transduction efficacy and toxicity and viral titers were determined using qPCR.

### PMN culture, nucleofection and viral transduction

PMN were prepared from embryonic day 14 Wistar rats as described previously.^64^ PMN were seeded in a density of 4 × 10^5^ neurons/cm^2^ on poly-L-ornithine/laminin-coated cover slips under serum-free conditions in PMN-medium composed of DMEM F-12 (Gibco, Life Technologies, Darmstadt, Germany), glucose, BSA, penicillin/streptomycin/neomycin, N1, glutamine and insulin (all from Sigma-Aldrich, Seelze, Germany). The average content of dopaminergic (TH-positive) neurons was 10% of all neurons; there were no glial cells in the culture as confirmed by GFAP and Iba1-stainings.^65^

PMN were transfected before seeding on DIV 1 with the given plasmids using nucleofection (Nucleofector II Device and Basic Primary Neurons Nucleofector Kit (VPI-1003), Lonza, Basel, Switzerland).^66^ For each experimental condition, a 90-*μ*l cell suspension containing 3.2–5.0 × 10^6^ neurons was nucleofected with 5 *μ*g plasmid DNA using the program G-013 according to the manufacturer's instructions. The EGFP group was transfected with 5 *μ*g p.EGFP, whereas the *α*Syn groups were co-transfected with p.EGFP (2 *μ*g) and p.*α*Syn (3 *μ*g) to allow for the later identification of *α*Syn-transfected neurons. Co-transfection rates of p.EGFP and p.*α*Syn were almost 100% (Supplementary Figure 3). Cells were then resuspended in PMN medium and cultured on 24-well plates (Sarstedt, Nümbrecht, Germany) in 500 *μ*l PMN medium per well at 37 °C and 5% CO_2_. At 3 h after nucleofection, two-third of the medium was exchanged to discard toxic substances from the nucleofection procedure. Further medium changes of half of the total medium per well were performed on DIV 1, 2 and 3. Cells were fixed or lysed for further analysis on DIV 5.

For virus transduction, AAV.synaptophysin-EGFP was added in a concentration of 1.5 × 10^7^ transforming units (TU) per well at 4 h after seeding and nucleofection. Further medium changes were performed as described above. Transduction rates of the AAV were 60% of all cells.

PMN were cultured for a total of 5 days until protein lysates for western blot or cell fixation for ICC were performed. For analysis of autophagic flux, 1 nM Bafilomycin (Sigma-Aldrich) was added to the medium 6 h before lysis in selected conditions.

### Antibodies, immunocytochemistry and western blot

The following antibodies were used for ICC and Western blot of PMN: Primary antibodies: mouse anti-human-*α*Syn monoclonal antibody (mAb) (Invitrogen, Carlsbad, CA, USA; Cat. No. 328100), 1 : 250; mouse anti-*α*Syn mAb clone 42 (BD Transduction Laboratories, Franklin Lakes, NJ, USA; Cat. No. 610786) (recognizing both human and rat *α*Syn), 1 : 250; rabbit anti-TH polyclonal antibody (pAb) (AB152, Millipore, Darmstadt, Germany), 1 : 250; mouse anti-*β*-tubulin mAb clone TUB 2.1 (Sigma-Aldrich), 1:500; mouse anti-GAPDH mAb clone 6C5 (Biotrend, Köln, Germany), 1 : 1000; mouse anti-LC3 mAb clone 5F10 (NanoTools, Teningen, Germany), 1 : 200; rabbit anti-p62 pAb (Sigma-Aldrich), 1 : 1000; secondary antibodies: goat anti-rabbit or -mouse IgGs conjugated with cy2, cy3 or horseradish peroxidase (Dianova, Hamburg, Germany) 1 : 1000; donkey anti-mouse cy5 (ab96878, Abcam, Cambridge, UK), 1 : 1000.

For ICC, PMN were fixed on cover slips in 4% PFA for 10 min at 4 °C, permeabilized for 10 min in aceton at −20 °C and blocked with Dako diluent (Dako, Glostrup, Denmark) for 20 min. Incubation in primary antibodies at 4 °C overnight was followed by incubation in secondary antibodies for 30 min at 37 °C. Cells were counterstained with DAPI and then embedded in Mowiol (Sigma-Aldrich).

For western blot, PMN were lysed in ice-cold 10 mM HEPES, 142 mM KCl, 5 mM MgCl, 2.1 mM EGTA, IGEPAL, protease and phosphatase inhibitor and dithiothreitol. Protein lysates were sonificated, resolved on SDS-PAGE and blotted on nitrocellulose membrane. After blocking with 5% milk for 1 h, the membrane was incubated in the primary antibody overnight at 4 °C. Then, horseradish peroxidase-coupled secondary antibodies were applied for 1 h at room temperature. Bands were visualized using enhanced chemiluminescence (ECL-solution: 250 mM Luminol, 90 mM p-coumaric acid, 1 M Tris-HCl, 30% hydrogen peroxide) and band intensities were analyzed with ImageJ 1.45 s software (open freeware provided by the NIH, Bethesda, MD, USA; http://imagej.nih.gov/ij/).

### Microscopy, evaluation of neurite morphology and autophagy

Bright-field and fluorescent images were taken on an Axioplan microscope equipped with a 16-bit greyscale CCD camera (AxioCam HRm) using AxioVision 4.6 software (Zeiss, Jena, Germany). For every coverslip, 10 adjacent pictures at × 20 magnification were taken along the diameter from one side to the other, to avoid a sampling bias. On the micrographs of the TH or EGFP fluorescence, the neurites were traced using the ImageJ plugin NeuronJ 1.4.2.^67^ For each neuron, the number of neurites originating from the soma, the individual length of each single neurite and the total sum length of all neurites of one neuron were determined.

A Sholl anlysis^36^ of single neurons was performed manually on the basis of the NeuronJ-traces. Forty-five randomly chosen neurons were analyzed per condition for each of three independent cultures, totaling *n*=135 neurons for each condition. The number of intersections of the neurite tree with increasing perimeters from the center of the soma was counted every 12.5 *μ*m up to a distance of 200 *μ*m. From these raw data, the critical value, the neurite maximum and the Schoenen ramification index were calculated.^37^

Micrographs of the LC3 ICC for analysis of macroautophagy were taken at × 63 with the pseudo-confocal microscope device ApoTome (Zeiss). The software ImageJ was used for quantification. The neuronal soma was selected in the EGFP stain using the 'freehand selections' tool. The soma selection was then transferred to the inverted LC3 picture ('restore selection'), which was thresholded to exclude background signals. The LC3 puncta per selected neuronal soma on the resulting image were then counted automatically using the 'analyze particle' function. The minimum size of an autophagic punctum was defined as 3 × 3 pixel, that is, 278 nm in diameter, based on the literature.^38^

All measurements were performed blinded. Results from at least three independent experiments were statistically evaluated using one-way ANOVA followed by Dunnett's *post hoc* test with significance at *P*<0.05.

### Colocalization analysis

PMN were transfected with the given plasmids using nucleofection as described above. Cells were fixed on DIV 5 with 8% PFA for 5 min at room temperature. For ICC, permeabilization in 0.3% Triton X100 for 5 min was followed by blocking in Dako diluent for 20 min and incubation in the following primary antibodies at 4 °C overnight: goat anti-LC3 pAb (Santa Cruz, Heidelberg, Germany, sc-16756), 1 : 50; mouse anti-human-*α*Syn mAb (Invitrogen; Cat. No. 328100), 1 : 500; rabbit anti-TH pAb (Zytomed systems, Berlin, Germany, Cat. No. 220-0694), 1 : 500. Incubation in the following secondary antibodies was performed for 15 min at 37 °C: donkey anti-goat Alexa 647, 1 : 1000, donkey anti-rabbit Alexa Fluor 546, 1 : 1000 (both from Invitrogen) and donkey anti-mouse Alexa Fluor 488 (Jackson Immunno-Research, Suffolk, UK), 1 : 500. Cells were finally embedded in Mowiol.

Confocal microscopy was performed at a Leica TCS SP5 (Leica, Wetzlar, Germany) equipped with LAS AF software version 2.6.3. Per cover slip, 10 TH- and *α*Syn-positive neurons were micrographed (63/1.4 numerical aperture oil objective, × 12 digital zoom, Airy 1, sequential scanning). The image files were exported in tif-format and opened with ImageJ 1.45 s. The soma of a TH-positive neuron (excluding nucleus and distal neurites) was selected on the TH-image using the 'freehand selection' tool. The 'create mask' command was applied to the selection and the resulting mask-image inverted. Using 'image calculator', the inverted mask was subtracted from both original LC3- and *α*Syn-channel pictures. This procedure resulted in two corresponding images containing the LC3 and *α*Syn signals localized specifically in the soma. Colocalization of LC3 and *α*Syn was evaluated on both images using JACoP plugin.^40^ Per condition, two cover slips from two independent cultures, respectively, were evaluated. Statistics were performed using one-way ANOVA followed by Tukey-Kramer *post hoc* test with significance at *P*<0.05.

### Evaluation of mitophagy

PMN were prepared and cultured as described above. Two hours after seeding, each well with 5 × 10^5^ neurons was transduced with 1.25 × 10^8^ TU AAV6 expressing EGFP only or co-expressing EGFP and *α*Syn-WT, -A30P or A53T under control of a synapsin promoter and enhanced by WPRE (same sequences as within the respective plasmids described above; AAV were a kind gift from Sebastian Kügler, Göttingen).^60, 62^ AAV6 were chosen owing to the good transduction efficacy of dopaminergic neurons^68^ resulting in equal *α*Syn expression levels among the groups that were checked before by western blot. Medium changes were performed on DIV 1 and DIV 4. PMN were fixed on DIV 5 with 8% PFA for 5 min at 37 °C. For ICC, cells were permeabilized in 0.3% Triton X100 for 5 min and blocked in DAKO diluent for 20 min. Incubation in the following primary antibodies was performed at 4 °C overnight: goat anti-LC3 pAb (sc-16756), 1 : 50; rabbit TOM20 pAb (sc11415), 1 : 50 (both from Santa Cruz) and mouse anti-TH mAb (Sigma, T1299), 1 : 500. Incubation in the following secondary antibodies was performed for 15 min at 37 °C: donkey anti-goat Alexa 647, 1 : 1000; donkey anti-rabbit Alexa Fluor 546, 1 : 1000 (both from Invitrogen) and donkey anti-mouse DyLight 405 (Dianova), 1 : 500. Cells were embedded in Mowiol.

Confocal microscopy was performed at the Leica TCS SP5 described above. The total number of LC3 puncta and the number of LC3 puncta colocalizing with TOM20 was counted manually in the soma of TH-positive neurons on at least 12 images per condition from three independent cultures in a blinded manner. The ratio of the two values was calculated and statistics were performed using one-way ANOVA with significance at *P*<0.05.

### Analysis of vesicle transport in PMN

To visualize intraneuronal vesicle transport, PMN were nucleofected with p.dsRed or co-nucleofected with p.dsRed and one of the *α*Syn variants p.*α*Syn-WT, -A30P or -A53T as described above. They were seeded on glass chamber slides to improve later imaging conditions. Four hours after seeding, 1.5 × 10^7^ TU of AAV.synaptophysin-EGFP were added. On DIV 5, PMN were transferred to a conditioned cell observation chamber (37 °C, 5% CO_2_) attached to an inverted microscope (Axiovert, Zeiss). Live imaging was performed at × 63 magnification. After identification of a nucleofected, that is, dsRed-positive neuron, pictures of synaptophysin-EGFP were taken every 500 ms for 10 s. The pictures were analyzed with the ImageJ plugin MultipleKymograph (http://www.embl.de/eamnet/ html/ body_kymograph.html). All measurements were performed blinded. Results from at least three independent experiments were statistically evaluated using one-way ANOVA followed by Dunnett's *post hoc* test with significance at *P*<0.05.

### Animal experiments

Animals were treated according to the regulations of the local animal research council and legislation of the State of Lower Saxony, Germany. For all experiments, adult female Wistar rats (200–300 g, Charles River, Wilmington, MA, USA) were used. All procedures (intravitreal virus injection, optic nerve live imaging) were performed under deep anesthesia with 10% ketamine (95 mg/kg body weight) and 2% xylazine (7 mg/kg body weight) injected intraperitoneally.

### Intravitreal virus injection and optic nerve live imaging

Live imaging of the optic nerve was performed 30 days after intravitreal AAV injection of 1 × 10^9^ transforming units AAV2/1 (3–5 *μ*l) as reported before.^44, 45^ AAV2/1 was chosen owing to its unique transduction efficacy in RGC.^44, 45^ In brief, the orbita of the deeply anesthesized animal was incised along the orbital rim and the lacrimal gland was partly removed. The eye bulb was slightly rotated by pulling the superior rectus muscle. After removing the retro-orbital connecting tissue and longitudinally incising the dura, the optic nerve was exposed. The rat was then transferred to a Zeiss Examiner microscope adapted for live imaging. After confirming the integrity of the EGFP-labeled axons, a crush lesion was performed by constricting a 10-0 polyamide suture (Ethicon, Johnson & Johnson Medical, Norderstedt, Germany) around the optic nerve at a distance of 2 mm from its insertion into the eye bulb for a duration of 30 s. Fluorescent pictures were taken of the area 500 *μ*m proximal and distal to the crush site before and 5 min after the crush and then every hour until 6 h after crush. Special care was taken to give optimal anesthesia and life support to the animal (constant usage of warming pad, measurement of heart rate and oxygen saturation, fluid substitution). After 6 h imaging, the animals were killed. Retinas were dissected and flat mounted in 30% glycerol to be examined for viral transduction efficacy, which was 30–50% of all RGCs on a regular basis.

A total number of six rats per group (three groups: AAV.EGFP, AAV.*α*Syn-WT and AAV.*α*Syn-A30P) was operated and imaged. Original z-stack images were projected to one plane using the function 'extended depth of focus' of the Zeiss Zen software. For each axon the AIR was calculated at all time points (*n*=15 axons per group). The AIR is defined as the sum length of axonal fragments divided by the total axon length before fragmentation. The length of axonal fragments was estimated with help of the ImageJ plugin NeuronJ.

### Optic nerve lysates for *α*Syn immunoblotting

Intravitreal injections were performed as described above (AAV.EGFP, AAV.*α*Syn-WT and AAV.*α*Syn-A30P; *n*=4 eyes per group). After 30 days, optic nerves were dissected and immediately frozen in liquid nitrogen. They were homogenized in lysis buffer (10 mM HEPES pH 7.2, 142 mM KCl, 5 mM MgCl_2_-6H_2_O, 1 mM EGTA, 1 mM DTT, 1% IGEPAL) supplemented with complete protease inhibitor and phosSTOP phosphatase inhibitor (Roche, Indianapolis, IN, USA). Lysates were centrifuged and supernatants were separated by SDS-PAGE and blotted on a nitrocellulose membrane. Membranes were incubated at 4 °C overnight in the following primary antibodies: anti-human-*α*Syn monoclonal antibody (mAb) (Invitrogen; Cat. No. 328100), 1 : 500; mouse anti-*α*Syn mAb clone 42 (BD Transduction Laboratories; Cat. No. 610786), 1 : 500; mouse anti-GAPDH mAb clone 6C5 (Biotrend, Köln, Germany), 1 : 2500. After washing, the membranes were incubated in horseradish peroxidase-secondary anitbodies (Dianova) for 1 h. The signal was visualized with ECL-solution and quantified with ImageJ 1.45 s software.

## Abbreviations

*α*Syn: alpha-Synuclein
A30P: Alanin to Prolin at amino-acid 30 point mutation of *α*Syn
A53T: Alanin to Threonin at amino-acid 53 point mutation of *α*Syn
AAD: Acute axonal degeneration
AAV: Adeno-associated viral vector
AIR: Axonal integrity ratio
ANOVA: Analysis of variance
CNS: Central nervous system
DIV: Day *in vitro*
EGFP: Enhanced green fluorescent protein
GAP43: Growth associated protein 43
ICC: Immunocytochemistry
LB: Lewy body
LC3: Microtubule-associated protein 1 light chain 3
mTOR: Mammalian target of rapamycin
PD: Parkinson's disease
PFA: Paraformaldehyde
PMN: Primary midbrain neurons
TH: Tyrosine hydroxylase
TOM20: Translocase of outer mitochondrial membrane 20 kDa subunit
TU: Transforming units
WT: Wildtype

## References

[bib1] 1Burke RE, O'Malley K. Axon degeneration in Parkinson's disease. Exp Neurol 2013; 246: 72–83. 22285449 10.1016/j.expneurol.2012.01.011 PMC3340476

[bib2] 2Orimo S, Uchihara T, Nakamura A, Mori F, Kakita A, Wakabayashi K et al. Axonal alpha-synuclein aggregates herald centripetal degeneration of cardiac sympathetic nerve in Parkinson's disease. Brain 2008; 131: 642–650. 18079166 10.1093/brain/awm302

[bib3] 3Braak H, Del Tredici K, Rub U, de Vos RA, Jansen Steur EN, Braak E. Staging of brain pathology related to sporadic Parkinson's disease. Neurobiol Aging 2003; 24: 197–211. 12498954 10.1016/s0197-4580(02)00065-9

[bib4] 4Braak H, Sandmann-Keil D, Gai W, Braak E. Extensive axonal Lewy neurites in Parkinson's disease: a novel pathological feature revealed by alpha-synuclein immunocytochemistry. Neurosci Lett 1999; 265: 67–69. 10327208 10.1016/s0304-3940(99)00208-6

[bib5] 5Galvin JE, Uryu K, Lee VM, Trojanowski JQ. Axon pathology in Parkinson's disease and Lewy body dementia hippocampus contains alpha-, beta-, and gamma-synuclein. Proc Natl Acad Sci USA 1999; 96: 13450–13455. 10557341 10.1073/pnas.96.23.13450 PMC23968

[bib6] 6Spillantini MG, Schmidt ML, Lee VM, Trojanowski JQ, Jakes R, Goedert M. Alpha-synuclein in Lewy bodies. Nature 1997; 388: 839–840. 9278044 10.1038/42166

[bib7] 7Bendor JT, Logan TP, Edwards RH. The function of alpha-synuclein. Neuron 2013; 79: 1044–1066. 24050397 10.1016/j.neuron.2013.09.004 PMC3866954

[bib8] 8Kruger R, Kuhn W, Muller T, Woitalla D, Graeber M, Kosel S et al. Ala30Pro mutation in the gene encoding alpha-synuclein in Parkinson's disease. Nat Genet 1998; 18: 106–108. 9462735 10.1038/ng0298-106

[bib9] 9Polymeropoulos MH, Lavedan C, Leroy E, Ide SE, Dehejia A, Dutra A et al. Mutation in the alpha-synuclein gene identified in families with Parkinson's disease. Science 1997; 276: 2045–2047. 9197268 10.1126/science.276.5321.2045

[bib10] 10Singleton AB, Farrer M, Johnson J, Singleton A, Hague S, Kachergus J et al. alpha-Synuclein locus triplication causes Parkinson's disease. Science 2003; 302: 841. 14593171 10.1126/science.1090278

[bib11] 11Giasson BI, Duda JE, Quinn SM, Zhang B, Trojanowski JQ, Lee VM. Neuronal alpha-synucleinopathy with severe movement disorder in mice expressing A53T human alpha-synuclein. Neuron 2002; 34: 521–533. 12062037 10.1016/s0896-6273(02)00682-7

[bib12] 12Lee MK, Stirling W, Xu Y, Xu X, Qui D, Mandir AS et al. Human alpha-synuclein-harboring familial Parkinson's disease-linked Ala-53 —> Thr mutation causes neurodegenerative disease with alpha-synuclein aggregation in transgenic mice. Proc Natl Acad Sci USA 2002; 99: 8968–8973. 12084935 10.1073/pnas.132197599 PMC124407

[bib13] 13St Martin JL, Klucken J, Outeiro TF, Nguyen P, Keller-McGandy C, Cantuti-Castelvetri I et al. Dopaminergic neuron loss and up-regulation of chaperone protein mRNA induced by targeted over-expression of alpha-synuclein in mouse substantia nigra. J Neurochem 2007; 100: 1449–1457. 17241127 10.1111/j.1471-4159.2006.04310.x

[bib14] 14Zhou W, Hurlbert MS, Schaack J, Prasad KN, Freed CR. Overexpression of human alpha-synuclein causes dopamine neuron death in rat primary culture and immortalized mesencephalon-derived cells. Brain Res 2000; 866: 33–43. 10825478 10.1016/s0006-8993(00)02215-0

[bib15] 15Gomez-Isla T, Irizarry MC, Mariash A, Cheung B, Soto O, Schrump S et al. Motor dysfunction and gliosis with preserved dopaminergic markers in human alpha-synuclein A30P transgenic mice. Neurobiol Aging 2003; 24: 245–258. 12498958 10.1016/s0197-4580(02)00091-x

[bib16] 16Lee HJ, Khoshaghideh F, Lee S, Lee SJ. Impairment of microtubule-dependent trafficking by overexpression of alpha-synuclein. Eur J Neurosci 2006; 24: 3153–3162. 17156376 10.1111/j.1460-9568.2006.05210.x

[bib17] 17Prots I, Veber V, Brey S, Campioni S, Buder K, Riek R et al. alpha-Synuclein oligomers impair neuronal microtubule-kinesin interplay. J Biol Chem 2013; 288: 21742–21754. 23744071 10.1074/jbc.M113.451815 PMC3724632

[bib18] 18Sousa VL, Bellani S, Giannandrea M, Yousuf M, Valtorta F, Meldolesi J et al. {alpha}-synuclein and its A30P mutant affect actin cytoskeletal structure and dynamics. Mol Biol Cell 2009; 20: 3725–3739. 19553474 10.1091/mbc.E08-03-0302 PMC2777932

[bib19] 19Takenouchi T, Hashimoto M, Hsu LJ, Mackowski B, Rockenstein E, Mallory M et al. Reduced neuritic outgrowth and cell adhesion in neuronal cells transfected with human alpha-synuclein. Mol Cell Neurosci 2001; 17: 141–150. 11161475 10.1006/mcne.2000.0923

[bib20] 20Danzer KM, Krebs SK, Wolff M, Birk G, Hengerer B. Seeding induced by alpha-synuclein oligomers provides evidence for spreading of alpha-synuclein pathology. J Neurochem 2009; 111: 192–203. 19686384 10.1111/j.1471-4159.2009.06324.x

[bib21] 21Szego EM, Gerhardt E, Kermer P, Schulz JB. A30P alpha-synuclein impairs dopaminergic fiber regeneration and interacts with L-DOPA replacement in MPTP-treated mice. Neurobiol Dis 2012; 45: 591–600. 22001606 10.1016/j.nbd.2011.09.017

[bib22] 22Liu G, Wang P, Li X, Li Y, Xu S, Ueda K et al. Alpha-synuclein promotes early neurite outgrowth in cultured primary neurons. J Neural Transm 2013; 120: 1331–1343. 23443897 10.1007/s00702-013-0999-8

[bib23] 23Vogiatzi T, Xilouri M, Vekrellis K, Stefanis L. Wild type alpha-synuclein is degraded by chaperone-mediated autophagy and macroautophagy in neuronal cells. J Biol Chem 2008; 283: 23542–23556. 18566453 10.1074/jbc.M801992200 PMC2527094

[bib24] 24Spencer B, Potkar R, Trejo M, Rockenstein E, Patrick C, Gindi R et al. Beclin 1 gene transfer activates autophagy and ameliorates the neurodegenerative pathology in alpha-synuclein models of Parkinson's and Lewy body diseases. J Neurosci 2009; 29: 13578–13588. 19864570 10.1523/JNEUROSCI.4390-09.2009 PMC2812014

[bib25] 25Friedman LG, Lachenmayer ML, Wang J, He L, Poulose SM, Komatsu M et al. Disrupted autophagy leads to dopaminergic axon and dendrite degeneration and promotes presynaptic accumulation of alpha-synuclein and LRRK2 in the brain. J Neurosci 2012; 32: 7585–7593. 22649237 10.1523/JNEUROSCI.5809-11.2012 PMC3382107

[bib26] 26Xilouri M, Vogiatzi T, Vekrellis K, Park D, Stefanis L. Abberant alpha-synuclein confers toxicity to neurons in part through inhibition of chaperone-mediated autophagy. PLoS One 2009; 4: e5515. 19436756 10.1371/journal.pone.0005515 PMC2677735

[bib27] 27Stefanis L, Larsen KE, Rideout HJ, Sulzer D, Greene LA. Expression of A53T mutant but not wild-type alpha-synuclein in PC12 cells induces alterations of the ubiquitin-dependent degradation system, loss of dopamine release, and autophagic cell death. J Neurosci 2001; 21: 9549–9560. 11739566 10.1523/JNEUROSCI.21-24-09549.2001 PMC6763041

[bib28] 28Yu WH, Dorado B, Figueroa HY, Wang L, Planel E, Cookson MR et al. Metabolic activity determines efficacy of macroautophagic clearance of pathological oligomeric alpha-synuclein. Am J Pathol 2009; 175: 736–747. 19628769 10.2353/ajpath.2009.080928 PMC2716969

[bib29] 29Winslow AR, Chen CW, Corrochano S, Acevedo-Arozena A, Gordon DE, Peden AA et al. alpha-Synuclein impairs macroautophagy: implications for Parkinson's disease. J Cell Biol 2010; 190: 1023–1037. 20855506 10.1083/jcb.201003122 PMC3101586

[bib30] 30Choubey V, Safiulina D, Vaarmann A, Cagalinec M, Wareski P, Kuum M et al. Mutant A53T alpha-synuclein induces neuronal death by increasing mitochondrial autophagy. J Biol Chem 2011; 286: 10814–10824. 21252228 10.1074/jbc.M110.132514 PMC3060532

[bib31] 31Chew KC, Ang ET, Tai YK, Tsang F, Lo SQ, Ong E et al. Enhanced autophagy from chronic toxicity of iron and mutant A53T alpha-synuclein: implications for neuronal cell death in Parkinson disease. J Biol Chem 2011; 286: 33380–33389. 21795716 10.1074/jbc.M111.268409 PMC3190914

[bib32] 32Ebrahimi-Fakhari D, Cantuti-Castelvetri I, Fan Z, Rockenstein E, Masliah E, Hyman BT et al. Distinct roles *in vivo* for the ubiquitin-proteasome system and the autophagy-lysosomal pathway in the degradation of alpha-synuclein. J Neurosci 2011; 31: 14508–14520. 21994367 10.1523/JNEUROSCI.1560-11.2011 PMC3587176

[bib33] 33Millecamps S, Julien JP. Axonal transport deficits and neurodegenerative diseases. Nat Rev Neurosc 2013; 14: 161–176. 10.1038/nrn3380 23361386

[bib34] 34Chu Y, Morfini GA, Langhamer LB, He Y, Brady ST, Kordower JH. Alterations in axonal transport motor proteins in sporadic and experimental Parkinson's disease. Brain 2012; 135: 2058–2073. 22719003 10.1093/brain/aws133 PMC4571141

[bib35] 35Jensen PH, Li JY, Dahlstrom A, Dotti CG. Axonal transport of synucleins is mediated by all rate components. Eur J Neurosci 1999; 11: 3369–3376. 10564344 10.1046/j.1460-9568.1999.00754.x

[bib36] 36Sholl DA. Dendritic organization in the neurons of the visual and motor cortices of the cat. J Anat 1953; 87: 387–406. 13117757 PMC1244622

[bib37] 37Schoenen J. The dendritic organization of the human spinal cord: the dorsal horn. Neuroscience 1982; 7: 2057–2087. 7145088 10.1016/0306-4522(82)90120-8

[bib38] 38Klionsky DJ, Abdalla FC, Abeliovich H, Abraham RT, Acevedo-Arozena A, Adeli K et al. Guidelines for the use and interpretation of assays for monitoring autophagy. Autophagy 2012; 8: 445–544. 22966490 10.4161/auto.19496 PMC3404883

[bib39] 39Li Q, Lau A, Morris TJ, Guo L, Fordyce CB, Stanley EF. A syntaxin 1, Galpha(o), and N-type calcium channel complex at a presynaptic nerve terminal: analysis by quantitative immunocolocalization. J Neurosci 2004; 24: 4070–4081. 15102922 10.1523/JNEUROSCI.0346-04.2004 PMC6729428

[bib40] 40Bolte S, Cordelieres FP. A guided tour into subcellular colocalization analysis in light microscopy. J Microsc 2006; 224: 213–232. 17210054 10.1111/j.1365-2818.2006.01706.x

[bib41] 41Vives-Bauza C, Zhou C, Huang Y, Cui M, de Vries RL, Kim J et al. PINK1-dependent recruitment of Parkin to mitochondria in mitophagy. Proc Natl Acad Sci USA 2010; 107: 378–383. 19966284 10.1073/pnas.0911187107 PMC2806779

[bib42] 42Iliev AI, Wouters FS. Application of simple photobleaching microscopy techniques for the determination of the balance between anterograde and retrograde axonal transport. J Neurosci Methods 2007; 161: 39–46. 17123628 10.1016/j.jneumeth.2006.10.010

[bib43] 43Knoferle J, Koch JC, Ostendorf T, Michel U, Planchamp V, Vutova P et al. Mechanisms of acute axonal degeneration in the optic nerve *in vivo*. Proc Natl Acad Sci USA 2010; 107: 6064–6069. 20231460 10.1073/pnas.0909794107 PMC2851885

[bib44] 44Koch JC, Knoferle J, Tonges L, Michel U, Bahr M, Lingor P. Imaging of rat optic nerve axons *in vivo*. Nat Protoc 2011; 6: 1887–1896. 22051801 10.1038/nprot.2011.403

[bib45] 45Koch JC, Tonges L, Barski E, Michel U, Bahr M, Lingor P. ROCK2 is a major regulator of axonal degeneration, neuronal death and axonal regeneration in the CNS. Cell Death Dis 2014; 5: e1225. 24832597 10.1038/cddis.2014.191 PMC4047920

[bib46] 46Kugler S, Lingor P, Scholl U, Zolotukhin S, Bahr M. Differential transgene expression in brain cells *in vivo* and *in vitro* from AAV-2 vectors with small transcriptional control units. Virology 2003; 311: 89–95. 12832206 10.1016/s0042-6822(03)00162-4

[bib47] 47Kanazawa T, Adachi E, Orimo S, Nakamura A, Mizusawa H, Uchihara T. Pale neurites, premature alpha-synuclein aggregates with centripetal extension from axon collaterals. Brain Pathol 2012; 22: 67–78. 21672073 10.1111/j.1750-3639.2011.00509.x PMC8029413

[bib48] 48Huang Y, Chegini F, Chua G, Murphy K, Gai W, Halliday GM. Macroautophagy in sporadic and the genetic form of Parkinson's disease with the A53T alpha-synuclein mutation. Transl Neurodegener 2012; 1: 2. 23210740 10.1186/2047-9158-1-2 PMC3506995

[bib49] 49McLean PJ, Kawamata H, Ribich S, Hyman BT. Membrane association and protein conformation of alpha-synuclein in intact neurons. Effect of Parkinson's disease-linked mutations. J Biol Chem 2000; 275: 8812–8816. 10722726 10.1074/jbc.275.12.8812

[bib50] 50Subramaniam SR, Vergnes L, Franich NR, Reue K, Chesselet MF. Region specific mitochondrial impairment in mice with widespread overexpression of alpha-synuclein. Neurobiol Dis 2014; 70: 204–213. 25016198 10.1016/j.nbd.2014.06.017 PMC4205109

[bib51] 51Chen L, Xie Z, Turkson S, Zhuang X. A53T human alpha-synuclein overexpression in transgenic mice induces pervasive mitochondria macroautophagy defects preceding dopamine neuron degeneration. J Neurosci 2015; 35: 890–905. 25609609 10.1523/JNEUROSCI.0089-14.2015 PMC4300331

[bib52] 52Chinta SJ, Mallajosyula JK, Rane A, Andersen JK. Mitochondrial alpha-synuclein accumulation impairs complex I function in dopaminergic neurons and results in increased mitophagy *in vivo*. Neurosci Lett 2010; 486: 235–239. 20887775 10.1016/j.neulet.2010.09.061 PMC2967673

[bib53] 53Yang ML, Hasadsri L, Woods WS, George JM. Dynamic transport and localization of alpha-synuclein in primary hippocampal neurons. Mol Neurodegener 2010; 5: 9. 20181133 10.1186/1750-1326-5-9 PMC2830200

[bib54] 54Li W, Hoffman PN, Stirling W, Price DL, Lee MK. Axonal transport of human alpha-synuclein slows with aging but is not affected by familial Parkinson's disease-linked mutations. J Neurochem 2004; 88: 401–410. 14690528 10.1046/j.1471-4159.2003.02166.x

[bib55] 55Saha AR, Hill J, Utton MA, Asuni AA, Ackerley S, Grierson AJ et al. Parkinson's disease alpha-synuclein mutations exhibit defective axonal transport in cultured neurons. J Cell Sci 2004; 117: 1017–1024. 14996933 10.1242/jcs.00967

[bib56] 56Katsuse O, Iseki E, Marui W, Kosaka K. Developmental stages of cortical Lewy bodies and their relation to axonal transport blockage in brains of patients with dementia with Lewy bodies. J Neurol Sci 2003; 211: 29–35. 12767494 10.1016/s0022-510x(03)00037-6

[bib57] 57Utton MA, Noble WJ, Hill JE, Anderton BH, Hanger DP. Molecular motors implicated in the axonal transport of tau and alpha-synuclein. J Cell Sci 2005; 118: 4645–4654. 16176937 10.1242/jcs.02558

[bib58] 58Siebert H, Kahle PJ, Kramer ML, Isik T, Schluter OM, Schulz-Schaeffer WJ et al. Over-expression of alpha-synuclein in the nervous system enhances axonal degeneration after peripheral nerve lesion in a transgenic mouse strain. J Neurochem 2010; 114: 1007–1018. 20524960 10.1111/j.1471-4159.2010.06832.x

[bib59] 59Perfeito R, Lazaro DF, Outeiro TF, Rego AC. Linking alpha-synuclein phosphorylation to reactive oxygen species formation and mitochondrial dysfunction in SH-SY5Y cells. Mol Cell Neurosci 2014; 62: 51–59. 25109238 10.1016/j.mcn.2014.08.002

[bib60] 60Tonges L, Szego EM, Hause P, Saal KA, Tatenhorst L, Koch JC et al. Alpha-synuclein mutations impair axonal regeneration in models of Parkinson's disease. Front Aging Neurosci 2014; 6: 239. 25309425 10.3389/fnagi.2014.00239 PMC4159996

[bib61] 61Koch JC, Barski E, Lingor P, Bahr M, Michel U. Plasmids containing NRSE/RE1 sites enhance neurite outgrowth of retinal ganglion cells via sequestration of REST independent of NRSE dsRNA expression. FEBS J 2011; 278: 3472–3483. 21790997 10.1111/j.1742-4658.2011.08269.x

[bib62] 62Taschenberger G, Garrido M, Tereshchenko Y, Bahr M, Zweckstetter M, Kugler S. Aggregation of alphaSynuclein promotes progressive *in vivo* neurotoxicity in adult rat dopaminergic neurons. Acta Neuropathol 2012; 123: 671–683. 22167382 10.1007/s00401-011-0926-8 PMC3316935

[bib63] 63Zolotukhin S, Byrne BJ, Mason E, Zolotukhin I, Potter M, Chesnut K et al. Recombinant adeno-associated virus purification using novel methods improves infectious titer and yield. Gene Ther 1999; 6: 973–985. 10455399 10.1038/sj.gt.3300938

[bib64] 64Lingor P, Unsicker K, Krieglstein K. Midbrain dopaminergic neurons are protected from radical induced damage by GDF-5 application. Short communication. J Neural Transm 1999; 106: 139–144. 10226934 10.1007/s007020050146

[bib65] 65Saal KA, Koch JC, Tatenhorst L, Szego EM, Ribas VT, Michel U et al. AAV.shRNA-mediated downregulation of ROCK2 attenuates degeneration of dopaminergic neurons in toxin-induced models of Parkinson's disease *in vitro* and *in vivo*. Neurobiol Dis 2014; 73: 150–162. 25283984 10.1016/j.nbd.2014.09.013

[bib66] 66Zeitelhofer M, Vessey JP, Thomas S, Kiebler M, Dahm R. Transfection of cultured primary neurons via nucleofection. Curr Protoc Neurosci 2009; Chapter 4: Unit 4.32. 10.1002/0471142301.ns0432s47 19340811

[bib67] 67Meijering E, Jacob M, Sarria JC, Steiner P, Hirling H, Unser M. Design and validation of a tool for neurite tracing and analysis in fluorescence microscopy images. Cytometry A 2004; 58: 167–176. 15057970 10.1002/cyto.a.20022

[bib68] 68Decressac M, Mattsson B, Lundblad M, Weikop P, Bjorklund A. Progressive neurodegenerative and behavioural changes induced by AAV-mediated overexpression of alpha-synuclein in midbrain dopamine neurons. Neurobiol Dis 2012; 45: 939–953. 22182688 10.1016/j.nbd.2011.12.013

