# Supplementary figures and images for: Alpha-Synuclein affects neurite morphology, autophagy, vesicle transport and axonal degeneration in CNS neurons
Source: Cell Death Dis. 2015 Jul 9;6(7):e1811–. doi: 10.1038/cddis.2015.169 (PMC4650722; doi:10.1038/cddis.2015.169)

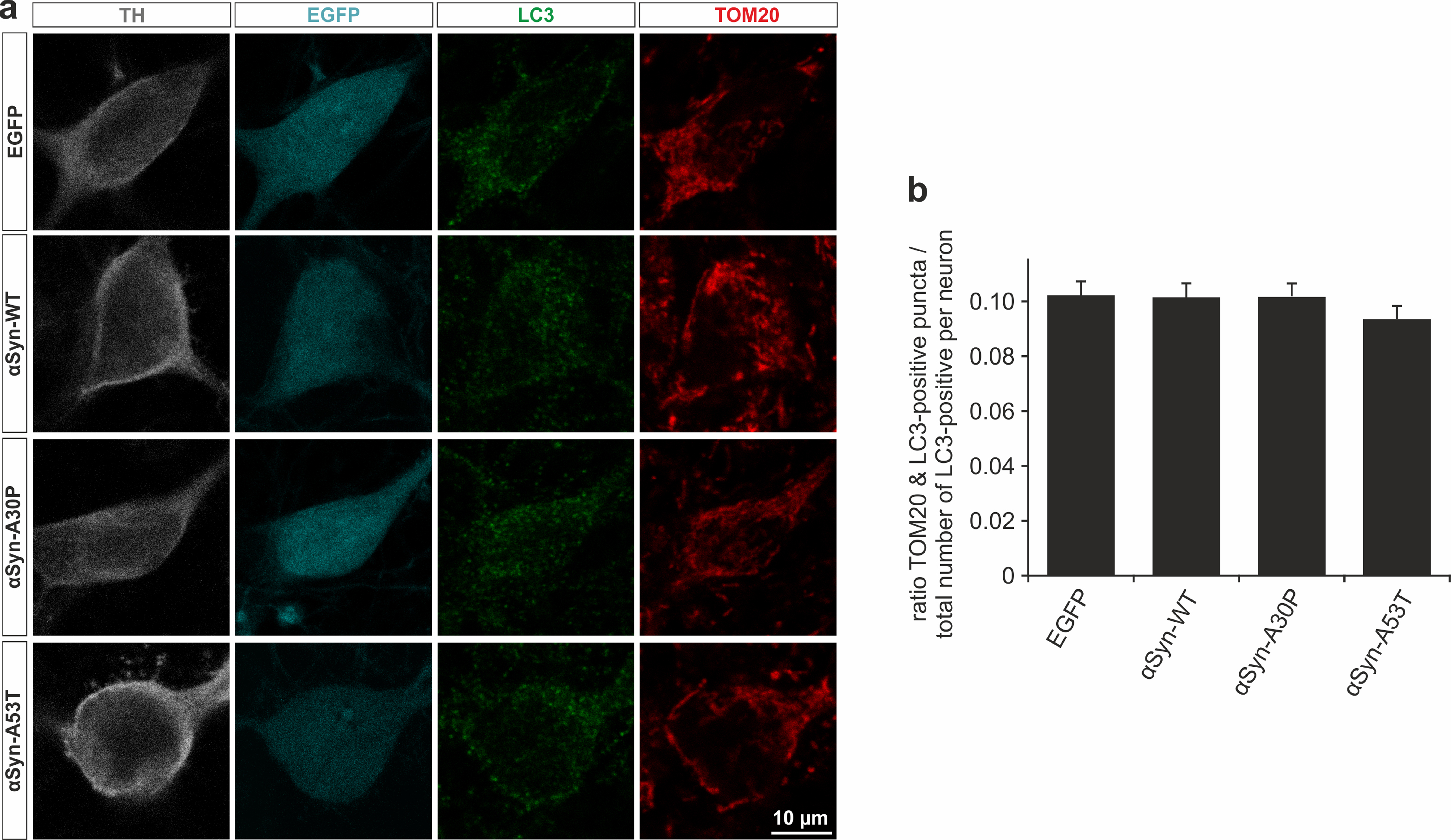

Supplement: Supplementary Figure 1 [file cddis2015169x1.tif]

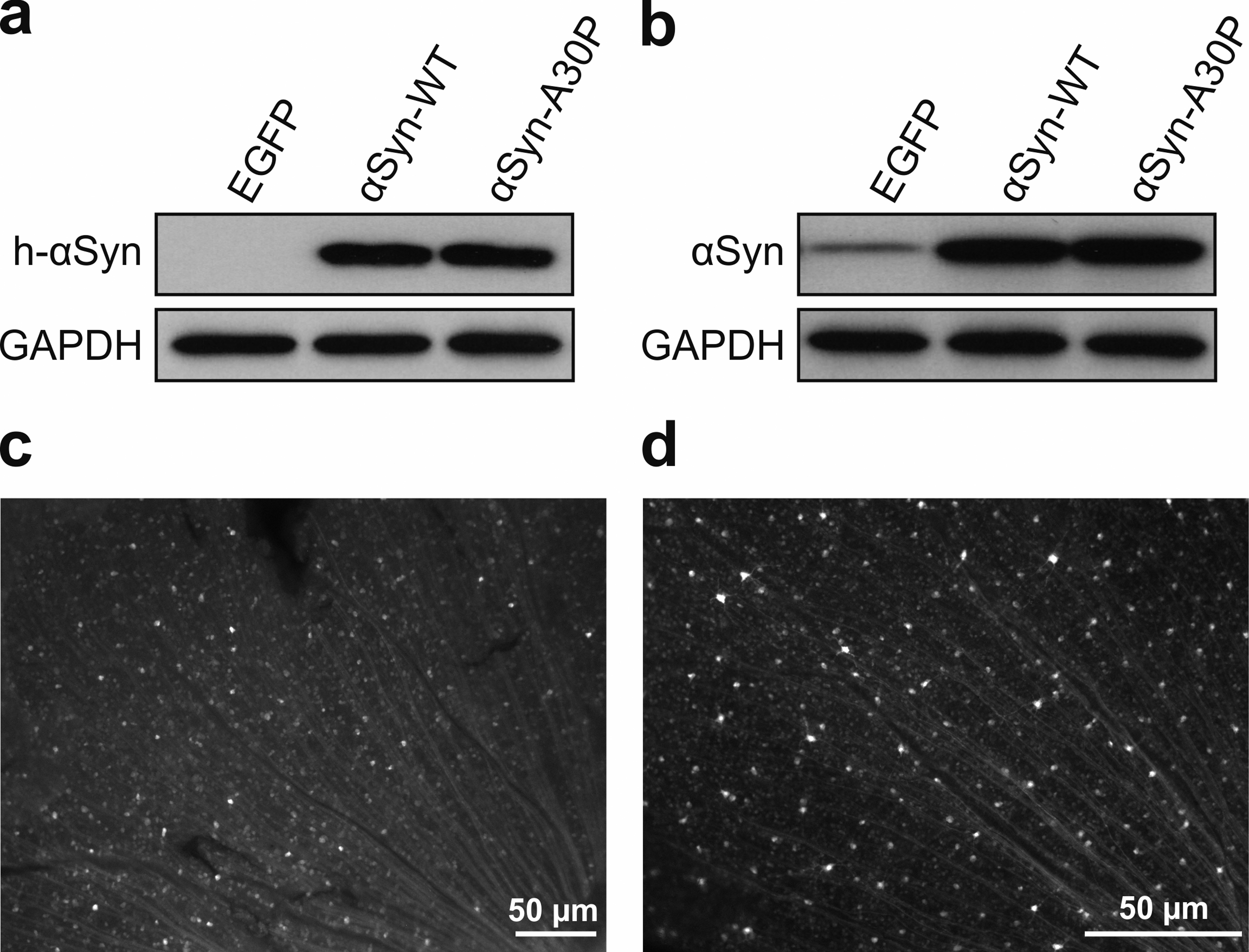

Supplement: Supplementary Figure 2 [file cddis2015169x2.tif]

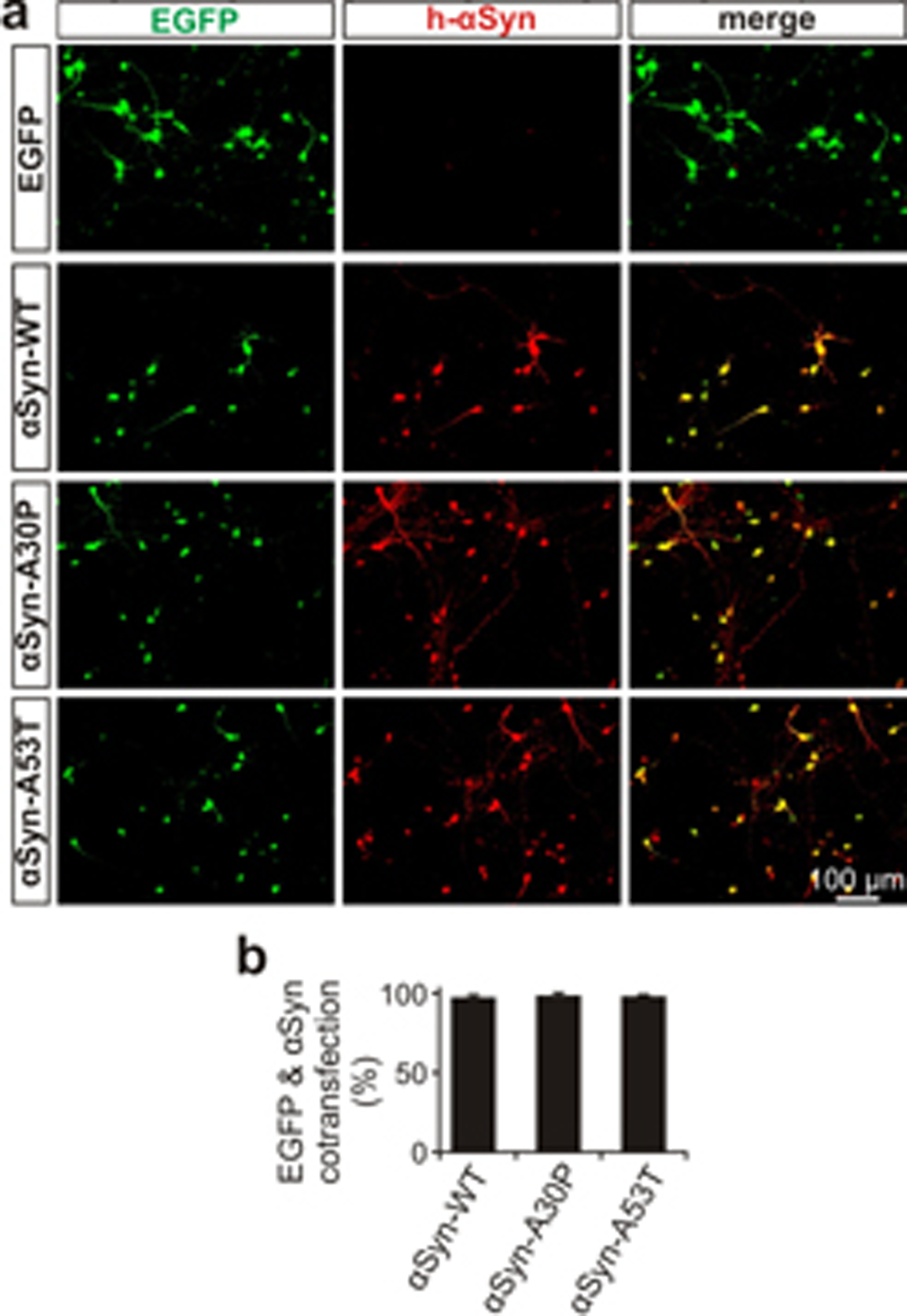

Supplement: Supplementary Figure 3 [file cddis2015169x3.tif]
