# Supplementary material for: Alpha-Synuclein affects neurite morphology, autophagy, vesicle transport and axonal degeneration in CNS neurons
Source: Cell Death Dis. 2015 Jul 9;6(7):e1811–. doi: 10.1038/cddis.2015.169 (PMC4650722; doi:10.1038/cddis.2015.169)
Supplement: Supplementary Figure Legends [file cddis2015169x4.doc]

**Supplementary Figure 1:** Colocalization of mitochondria and LC3 in PMN.

(**a**) Representative confocal micrographs of PMN transduced with EGFP alone or EGFP and the respective αSyn-variant indicated on the left side and immunostained against TH, LC3 and the mitochondrial marker TOM20 as specified on top.

(**b**) The amount of mitophagy after transfection with different αSyn-variants was quantified by counting the number of LC3-puncta co-staining with TOM20 in each TH-positive neuron. This value was then divided by the total number of LC3-puncta per cell to obtain the given ratio that did, however, not show significant differences among the groups.

Statistics: One-way ANOVA with significance level p<0.05. Error bars represent means ± SEM. n=4 independent experiments.

**Supplementary Figure 2:** AAV-mediated overexpression of αSyn in the optic nerve.

(**a,b**) Immunoblots of rat optic nerve lysates 4 weeks after intravitreal injection of AAV expressing EGFP alone or EGFP and the given αSyn-variant. In (a), an antibody specific for human αSyn (Invitrogen) was used to detect only the human αSyn expressed by the injected AAV. In (b), total αSyn levels including endogenous expression (which is naturally rather low in the optic nerve) were assessed using an antibody recognizing both human and rat αSyn (BD).

(**c,d**) Representative micrographs of flat mounted retinas at 4 weeks after intravitreal injection of AAV.αSyn-WT (c) or AAV.EGFP (d) showing in green fluorescence the high transduction efficacy of the EGFP-expressing viral vectors. Retina flat mounts were obtained after every live imaging and showed transduction rates of 30-50% of all RGCs on a regular basis.

**Supplementary Figure 3:** Assessment of co-transfection efficacy.

(**a,b**) PMN were electroporated with a total amount of 5 µg DNA, either with 5 µg p.EGFP alone or co-transfected with 2 µg p.EGFP and 3 µg p.αSyn-WT, p.αSyn-A30P or p.αSyn-A53T. Representative pictures of an αSyn-immunocytochemistry for the plasmids given on the left side are shown in (a). Co-transfection rates of EGFP and the αSyn-plasmids were almost 100% as quantified in (b). Statistics: One-way ANOVA followed by Dunnett’s post-hoc test, **: p<0.005,***: p<0.0005. Error bars represent means ± SEM.
